# New Appliances and Things Medical

**Published:** 1901-11-16

**Authors:** 


### NEW APPLIANCES AND THINGS MEDICAL.

[We shall be glad to receive, at our Office, 28 & 29 Southampton Street, Strand, London, W.C., from the manufacturers, specimens of all new preparations and appliances which may be brought out from time to time.]

### PATENT WATERPROOF BED-SHEETING.

(W. S. Rothband and Co., Premium Rubber Works, 61 Elizabeth Street, Cheetham, Manchester.)

This new improved waterproof sheeting has many advantages which will commend themselves to those who have charge of sick persons. Though waterproof, it is not made of indiarubber, and when put away and kept for considerable periods of time it does not stick together nor crack. Further, it will resist the action of most chemical bodies, including turpentine, alcohol, chloroform, and what is most important, soap and water. The price is a further consideration, being almost the same as ordinary rubber sheeting, with infinitely better lasting qualities. The width is 36 inches, and the price 3s. 6d. per yard in large quantities, 3s. 9d. when shorter lengths have to be cut to order. We thoroughly recommend this new sheeting to the authorities of hospitals, workhouses, and other institutions.

### THE IDEAL BANDAGE.

(Sole London Agent: M. Sello, 12a Finsbury Square, London, E.C.)

The theoretical advantages of this new bandage are fully confirmed by practical experience of its use. For several years the writer has employed Crêpe Velpeau bandages in his practice, and has discarded all others, and in his opinion there is no fault to find with them; they are practically perfect. They are, however, extremely expensive, and soon lose their elasticity, entailing constant renewal when it is necessary to maintain an equable pressure. Against the new Ideal bandages these objections cannot be urged; they can be produced by the manufacturer and sold by the chemist at a price which compares very favourably with that of anything else of the sort, and not only do they retain their elasticity but suffer little or no impairment of the latter quality by the process of washing with soap and water. They are made of pure cotton and have a large range of elasticity. A special variety is linted on one surface, and can be applied with the greatest ease without having recourse to any "turns," thus lies smoothly and exceedingly comfortable. According to the method of rolling these bandages it is apparently intended that the linted side should be on the outside. From our limited experience we should have thought that by applying them with the linted side next the skin the bandages would cling more closely, look neater, and collect less dirt on the outer surface This is, however, a minor detail, and by re-rolling before use this objection, if it be an objection, can be at once rectified.

### ADRENALIN CHLORIDE AND BORO-CHLORETONE.

(Park, Davis and Co., 111 Queen Victoria Street, London, E.C.)

The therapeutic effect of extract of adrenal gland substance has become familiar in practice, and especially is it valuable in those cases in which the adrenal glands are known to be functionless or functionally inadequate. It is only recently, however, that the active principle of these glands has been isolated and prepared in a commercial form. Adrenalin chloride, as supplied by Park, Davis and Co., is a solution of the strength of 1 part in 1,000 of normal salt solution. As now prepared by this firm it is a permanent solution, a great triumph of pharmaceutical art. For certain diseases of the nose, such as hay fever, a solution of this kind will be much appreciated by the profession, and for Addison's disease as far as we know at present it is the only therapeutic agent which can be regarded as rational. Boro-chloretone is a new dusting powder which has valuable properties, for, in addition to the fact that the powder is exceedingly finely pulverised and hence non-irritating, it possesses antiseptic and anæsthetic properties which render it valuable in the treatment of painful and septic ulcers. Chloretone itself has such distinct advantages as a local anæsthetic in addition to those of a general hypnotic, that it deserves to be better known, and its therapeutic properties more appreciated than is at present the case in this country.

